# Conservative Tests under Satisficing Models of Publication Bias

**DOI:** 10.1371/journal.pone.0149590

**Published:** 2016-02-22

**Authors:** Justin McCrary, Garret Christensen, Daniele Fanelli

**Affiliations:** 1 School of Law, University of California, Berkeley, California, United States of America; 2 National Bureau of Economic Research (NBER), Cambridge, Massachusetts, United States of America; 3 Berkeley Initiative for Transparency in the Social Sciences (BITSS), University of California, Berkeley, California, United States of America; 4 Berkeley Institute for Data Science (BIDS), University of California, Berkeley, California, United States of America; 5 Meta-Research Innovation Center at Stanford (METRICS), Stanford University, Stanford, California, United States of America; Universiteit Gent, BELGIUM

## Abstract

Publication bias leads consumers of research to observe a selected sample of statistical estimates calculated by producers of research. We calculate critical values for statistical significance that could help to adjust after the fact for the distortions created by this selection effect, assuming that the only source of publication bias is file drawer bias. These adjusted critical values are easy to calculate and differ from unadjusted critical values by approximately 50%—rather than rejecting a null hypothesis when the t-ratio exceeds 2, the analysis suggests rejecting a null hypothesis when the t-ratio exceeds 3. Samples of published social science research indicate that on average, across research fields, approximately 30% of published t-statistics fall between the standard and adjusted cutoffs.

## Introduction

A natural tendency in scientific work is for statistically significant results to be reported with greater likelihood than insignificant results.

In fields like economics or psychology, where hypothesis testing plays an important role in establishing the robustness of estimated effects, this tendency may result in a systematic selection effect, whereby published estimates are more extreme than the underlying population effects. Rosenthal famously notes that in the “extreme view of this problem, the ‘file drawer problem,’ …the journals are filled with the 5% of the studies that show type I errors, while the file drawers back at the lab are filled with the 95% of the studies that show nonsignificant …results.” (See p. 638 in [1].) This fundamental problem has been widely acknowledged and appreciated in economics [2–13]. In recent years the issue has received renewed attention in finance [14–18], in statistics [19–22], in political science [23, 24], in psychology [1, 25–27], in medicine [28–30], and in other fields. Publication bias in medicine is a sufficiently serious concern that the U.S. Congress first mandated trial registration in 1997, the clinicaltrials.gov trial registry was created in 2000, expanded in 2007, and the NIH recently sought public comment on a further expansion of the requirements of trial results reporting [31].

If a file drawer bias leads statistically significant results to be more prevalent in the published literature than they would be in the absence of this bias, then the threshold for the results of statistical tests to be significant should be higher than it otherwise would be to maintain the desired type I error rate. This intuitive idea motivates the analysis of this paper. We show that if a finding’s conditional publication probability given the result of a statistical test is a step function, with step occurring at the conventional critical value for the test—in other words, a satisficing model of publication bias—then an adjustment to conventional critical values restores the intended type I error rate (significance level) of the test among the sample of published papers. These adjusted critical values are simple to calculate and require only access to a table of the values of the original test statistic.

We give examples of adjusted critical values for various test statistics under conventional levels of significance. These adjusted critical values are an average of 49% larger than the corresponding unadjusted critical values. For example, if authors use two-tailed t-tests to gauge the robustness of their findings, and only submit findings with t-test values above 1.96 in absolute value, then 5 percent of the t-tests observed by the editor will exceed 3.02 in absolute value. An editor seeking to counteract the selection effect created by authors’ behavior, then, would use a critical value of 3.02. Further, suppose a literature contains independent estimates of the same quantity, with authors testing the same null hypothesis using the t-test, and that 95 percent of the t-statistics in the literature are between 2 and 3 in absolute value, with the remainder above. Despite this (hypothetical) literature of significant results, there would in fact be little evidence against the null hypothesis, if file drawer bias prevented the submission of insignificant estimates. (Naturally, this game between authors and editors can be interpreted to apply equally to editors and readers, with readers (editors) playing the role of the editors (authors).)

The approach taken here in addressing publication bias is to restore the intended type I error rate of hypothesis tests by adjusting the critical value. The approach we propose might help to assess the reliability of an existing literature, and could complement current methods to assess and correct retrospectively for publication bias. Current approaches typically apply to meta-analysis and make use of funnel plots and related meta-regression techniques aimed at estimating the possible presence of file-drawer effects and recovering point estimates for the population average of the underlying estimates [32–34]. These latter methods are relatively narrow in scope and are sensitive to deviations from their underlying assumptions, for example by requiring large sample sizes and low heterogeneity [35].

Researchers have addressed the issue of hypothesis testing in the context of publication bias [15], but have focused on selection rules, such as specification searching and data mining, that are more pernicious than satisficing models. For the problems created by file drawer bias, a satisficing model may be realistic. Other types of publication bias may not be consistent with satisficing models, and the results of this paper do not apply to such problems. One way to understand the contribution of the present paper is that it clarifies that a researcher who insists on truly decisive rejections of null hypotheses (e.g., t-tests greater than 5 in absolute value) must implicitly believe in more troublesome forms of publication bias than simple file drawer bias (setting aside issues regarding inappropriate standard error calculation).

## Methods

Suppose authors calculate a test statistic, *T*, and plan to reject at the 1 − *α* percent level a given null hypothesis if *T* > *c*_1−*α*_, for a known critical value *c*_1−*α*_. For example, *T* could be the square of the t-ratio for a regression coefficient and authors could plan to reject the null hypothesis of zero if *T* exceeds *c*_0.95_ = 1.96^2^. Let the distribution function of *T* under the null hypothesis be denoted *F*(⋅) and let *F*^−1^(⋅) denote the corresponding quantile function. (Throughout, we will assume that the quantile function is uniquely defined, i.e., *F*(⋅) is strictly monotonic. We also assume the null hypothesis is true, though this is also the case with all regularly calculated p-values and test statistics.) The critical value *c*_1−*α*_ is given by *F*^−1^(1 − *α*), because then the probability of false rejection is *P*(*T* > *c*_1−*α*_) = 1 − *F*(*F*^−1^(1 − *α*)) = *α*.

Throughout, we assume that authors submit statistically insignificant results with probability *π*_0_, but submit statistically significant results with probability *π*_1_. Formally, we state

Assumption 1.

$$\begin{array}{c} P\left(D=1|T\right)=\pi_{0}1\left(T\leq c_{1-\alpha }\right)+\pi_{1}1\left(T>c_{1-\alpha }\right) \end{array}$$

where *D* equals one if a study is submitted and equals zero otherwise. Thus, the conditional probability of submission is a step function, with step occurring at *c*_1−*α*_ and with step height *π*_1_ − *π*_0_. A few remarks are in order. First, Assumption 1 would be unreasonable if different individuals had differing views regarding the significance level at which tests should be conducted. However, as there is a clear default of *α* = 0.05, the assumption seems reasonable. Second, while Assumption 1 simplifies the analysis, it is not the only condition under which the results derived in the next section obtain. In particular, it is not important that the conditional probability be constant to the left of *c*_1−*α*_. However, it is important that it be constant to the right of *c*_1−*α*_. That the submission probability be constant to the right of *c*_1−*α*_ is in fact the essence of a satisficing model of publication bias: there exists a threshold at which an estimate becomes statistically significant, and authors are just as likely to submit a paper with a test statistic of *T* = *c*_1−*α*_ + *a* as they are to submit a paper with a test statistic of *T* = *c*_1−*α*_ + *b*, for *b* > *a* > 0.

## Results

Under Assumption 1, the distribution function of submitted test statistics is given by

$$\begin{array}{c} G\left(t\right)=\{\begin{array}{cc} \frac{\pi_{0}}{\pi }F\left(t\right) & \text{if}t\leq c_{1-\alpha }\\ 1-\frac{\pi_{1}}{\pi }\left(1-F\left(t\right)\right) & \text{if}t>c_{1-\alpha } \end{array} \end{array}$$ (1)

where *π* is the unconditional probability of submission: *π* = *απ*_1_ + (1 − *α*)*π*_0_. The calculation is a straightforward application of Bayes’ rule, as follows: define *G*(*t*) = *P*(*T* ≤ *t*|*D* = 1). Bayes’ rule implies $G\left(t\right)=\frac{P(D=1|T\leq t)P(T\leq t)}{P(D=1)}$. In the first case, fix *t* ≤ *c*_1−*α*_. Substituting, we find $G\left(t\right)=\frac{\pi_{0}}{\pi }F\left(t\right).$ In the second case, fix *t* > *c*_1−*α*_. *G*(*t*) = 1 − *P*(*T* > *t*|*D* = 1). Bayes implies $1-P\left(T>t|D=1\right)=1-\frac{P(D=1|T>t)P(T>t)}{P(D=1)}$. Substituting, we have $G\left(t\right)=1-\frac{\pi_{1}}{\pi }\left(1-F\left(t\right)\right)$, as above. Note that Eq (1) implies a test of Assumption 1 that is implementable using meta-analytic data, as by Card and Krueger [8].

By inverting *G*(⋅), we can derive a formula for critical values that adjust for type I error rate distortions induced by file drawer bias.

Lemma. *G*^−1^(1 − *α*) = *F*^−1^(1 − *απ*/*π*_1_) = *c*_1−*απ*/*π*_1__

Proof. For every *t*, *G*(*t*) ≤ *F*(*t*). Therefore, *G*^−1^(1 − *α*) ≥ *c*_1−*α*_, and we have

$$\begin{array}{c} 1-\alpha =G\left(d_{1-\alpha }\right)=1-\frac{\pi_{1}}{\pi }(1-F(d_{1-\alpha }))\;\;\Leftrightarrow \;\;F\left(d_{1-\alpha }\right)=1-\alpha \pi /\pi_{1} \end{array}$$

where *d*_1−*α*_ ≡ *G*^−1^(1 − *α*).

The lemma clarifies that to undo the selection effect created by authors’ selective submission, an editor should calculate the critical value for the relevant testing procedure, using any standard table for the test, but pretending that the desired type I error rate was *απ*/*π*_1_. Under the null hypothesis and Assumption 1, such a procedure will guarantee a testing procedure with type I error rate *α*. (One could choose any type I error rate here, not just *α*, as this refers to only the type I error rate among the submitted test statistics, which is clearly a selected sample. We choose *α* for its intuitive appeal, keeping the level of false positives identical across the entire universe of tests and the selected sample of submitted tests.)

This conclusion would seem to be of little practical consequence, since neither *π*_1_ nor *π*_0_ are known. However, it is straightforward to derive bounds under a worst-case scenario.

Proposition. Under the null hypothesis and Assumption 1, a test with type I error rate no more than *α* is obtained by utilizing a critical value of *F*^−1^(1 − *α*^2^).

Proof. Since *G*(⋅) is increasing in *π*_0_, an upper bound on the critical value is obtained by setting *π*_0_ = 0. Since *G*^−1^(1 − *α*) > *F*^−1^(1 − *α*), we have

$$\begin{array}{c} 1-\alpha =G\left(d_{1-\alpha }^{*}\right)=1-\frac{1}{\alpha }(1-F(d_{1-\alpha }^{*}))\;\;\Leftrightarrow \;\;F\left(d_{1-\alpha }^{*}\right)=1-\alpha^{2} \end{array}$$

where $d_{1-\alpha }^{*}\equiv \sup_{\pi_{0},\pi_{1}}G^{-1}\left(1-\alpha \right)$.

### Examples

Table 1 lists unadjusted and adjusted critical values for selected tests, where the adjusted critical values are those of the proposition. As discussed, under a satisficing model of publication bias, use of these critical values guarantees that the tests have type I error rate of at most 5 percent. The tests considered are two-tailed t-tests, F-tests with 5 and 10 numerator degrees of freedom and a large denominator degrees of freedom, and two types of nonparametric two-sample tests (Kolmogorov-Smirnov and Feller). All tests are of the form “reject if *T* > *c*_1−*α*_, ” for some *T* and some *c*_1−*α*_.

**Table 1:** Unadjusted and Adjusted Critical Values, Selected Testing Procedures.

Standard t-test				
Type I Error Rate		Unadjusted	Adjusted	
0.1		1.64	2.58	
0.05		1.96	3.02	
0.01		2.58	3.89	
F-test				
	5 numerator d.o.f.		10 numerator d.o.f.	
Type I Error Rate	Unadjusted	Adjusted	Unadjusted	Adjusted
0.10	1.85	3.02	1.60	2.32
0.05	2.21	3.68	1.83	2.71
0.01	3.02	5.15	2.32	3.56
Two-Sample Tests				
	Kolmogorov-Smirnov		Feller	
Type I Error Rate	Unadjusted	Adjusted	Unadjusted	Adjusted
0.10	1.23	1.63	1.07	1.52
0.05	1.36	1.83	1.22	1.73
0.01	1.63	2.23	1.52	2.15

Note: Table reports critical values, unadjusted and adjusted, for selected commonly utilized testing procedures. Entries for t-test are absolute values of critical values. Entries for F-test are for denominator degrees of freedom equal to 100,000 See text for details.

The t- and F-test distributions are standard [36] and critical values are calculated using statistical software. The Kolmogorov-Smirnov two-sample test statistic is given by $T=\sqrt{n}\sup_{x}|{\hat{H}}_{1}\left(x\right)-{\hat{H}}_{2}\left(x\right)|$ where ${\hat{H}}_{1}\left(x\right)$ and ${\hat{H}}_{2}\left(x\right)$ are the empirical distribution functions for two independent samples of sizes *n*_1_ and *n*_2_ drawn, under the null hypothesis, from the same distribution, and where *n* = *n*_1_ *n*_2_/(*n*_1_ + *n*_2_). Under the null, the distribution function for *T* converges to $t\mapsto 1-2\sum_{j=1}^{\infty }{\left(-1\right)}^{j-1}\exp\left(-j^{2}t^{2}\right)$ (critical values for this distribution are taken from the tabulation in [37]). The Feller two-sample test statistic is $T=\sqrt{n}\sup_{x}\{{\hat{H}}_{1}\left(x\right)-{\hat{H}}_{2}\left(x\right)\}$, with limiting distribution function of *t* ↦ 1 − exp(−2*t*^2^) (Theorem 4, [38]). Critical values for this distribution are calculated directly.

Looking over Table 1, it is apparent that the adjusted critical values for the tests considered are 30 to 70 percent larger than the corresponding unadjusted critical values. For example, the adjusted critical values for the common t-test are about 50 percent larger than their unadjusted counterparts. For a test of 5 percent type I error rate, we reject the null hypothesis if the absolute value of the t-ratio exceeds 1.96. Adjusting for file drawer bias, we reject if it exceeds 3.02. Adjusted critical values for the F-test with 10 numerator degrees of freedom are similarly about 50 percent larger than their unadjusted counterparts, while those for the F-test with 5 numerator degrees of freedom are 60-70 percent bigger.

### Statistics from Published Papers

**Fig 1:**
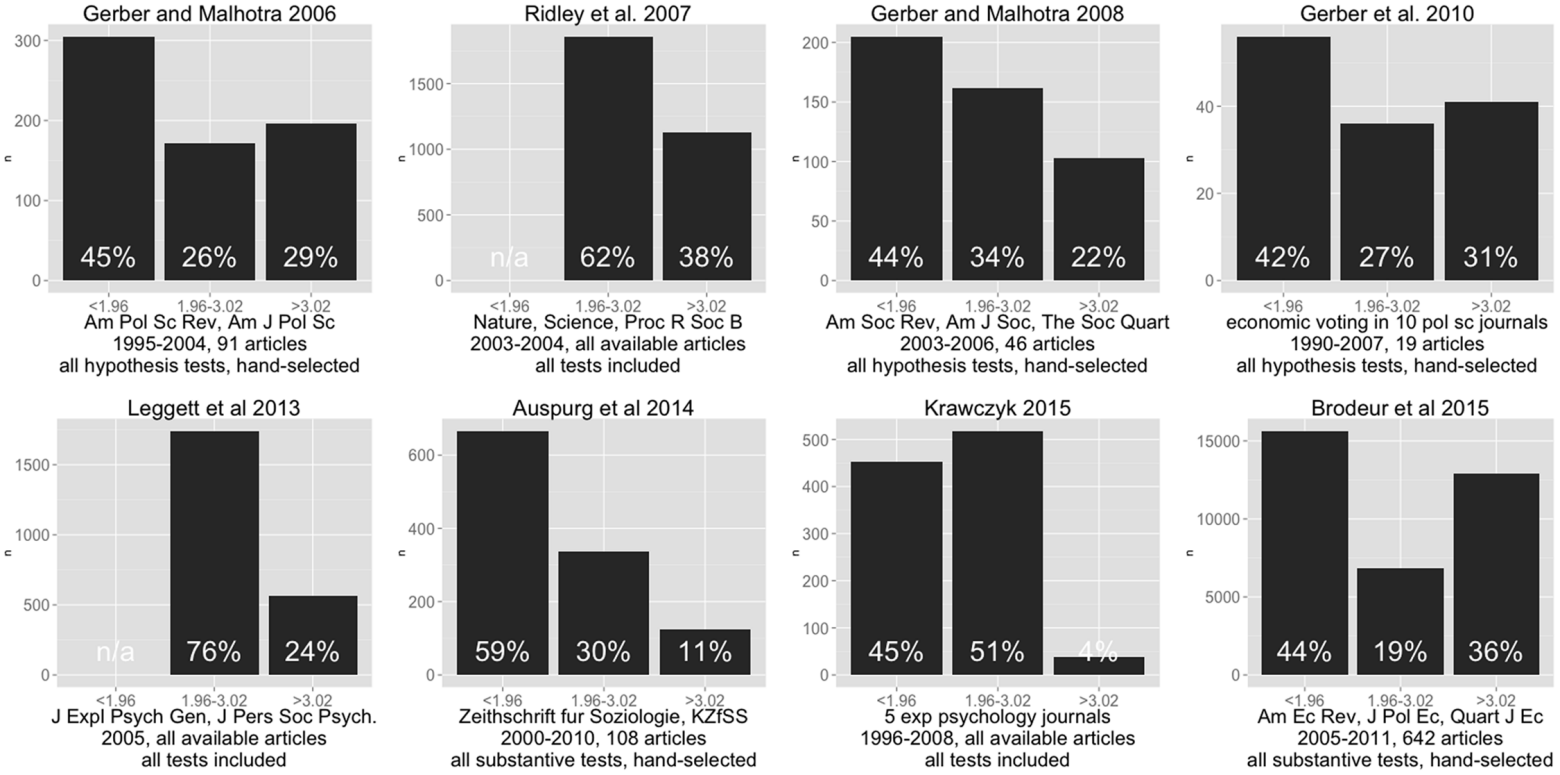
Distribution of t-statistics in Research Literature. Figure shows the distribution of t-statistics, as reported in the literature, that would lie below, between, or above the non-adjusted and adjusted threshold. Data were obtained from independent publications, which are referenced above each graph, and were either provided by the original authors or were re-digitized from histograms provided in the texts. Below each graph are indicated the following key methodological characteristics: study sampling strategy (i.e. specific journals or specific field), year range, number of articles included, and selection strategy for the statistics (i.e. whether, from each of the included articles, the authors had taken all available statistics, only those referring to explicit hypotheses, or only a subsample of “substantive” results selected by human coders).

To gauge the practical difference in published research between standard and adjusted cutoffs, we probed the literature for data on the distribution of t-statistics to be compared to the adjusted and unadjusted cutoffs for two-tailed tests at the *α* = .05 level. Our search yielded eight studies with publicly available data (or data available in the original figures themselves) in the behavioral and social sciences and one from biology that assessed the prevalence of the file-drawer problem by examining the distribution of t-values or equivalent statistics [13, 39–45]. These studies differed widely in key methodological choices including: discipline, sampling strategy (e.g. some surveyed specific journals, others specific topics), sample size, historical period considered, and in whether they had extracted all available statistics or hand-selected substantive ones. This heterogeneity precludes the calculation of a meta-analytical summary. Nonetheless, this collection of published results yields a relatively coherent picture by suggesting that, in the behavioral and social sciences, on average across studies 31% of all test statistics might lie between the adjusted and unadjusted cutoffs (N studies = 6, excluding two studies which lacked data for t-statistics below 1.96) and 22% lie above the adjusted cutoff (N = 6, range: 4%-36%). Looking only at the fraction of test statistics above the standard threshold, across studies an average of 62% lie between the adjusted and unadjusted cutoffs (N = 8) and an average of 38% lie above the adjusted cutoff (N = 8, range: 7%-65%) (Fig 1).

## Discussion

Intuitively, the results reported here are due to the narrow tails of the asymptotic distribution of most test statistics. For example, most econometric test statistics are asymptotically normal. The tails of the normal distribution decline so rapidly that, even if only significant results are observed, the chances are still quite good that a randomly chosen draw above the critical value will be close to the critical value. Thus, adjustments for the types of publication bias discussed in this paper may be small.

Empirically, however, it appears that a quite sizable fraction of published research that appears significant by normal standards would not meet the adjusted standard of our satisficing model. Notably, this evidence comes mainly from the social sciences, where significance testing is common and where we have been able to find reasonably sized samples of published research. We conjecture that our method would make a modest difference in fields that typically work with large data, whilst it might have a significant impact in fields in which small sample sizes and low signal-to-noise ratios are common. This conjecture is supported by the data we have gauged from the literature: t-statistic distributions from psychology—a discipline that is believed to have relatively low reproducibility rates (see [46])—tend to have a higher concentration in the 1.96-3.02 range compared to other social sciences.

The results obtained in this paper are highly specific in at least a few other regards. First, the results in this paper do not apply to settings where *F*(⋅) fails to be the distribution function of the test statistic in question. This would occur, for example, in settings where regression standard errors are calculated incorrectly [47–49].

Second, they do not pertain to specification searching. For example, suppose we model specification searching in the following way. Imagine that authors estimate *J* independent models, where the discrepancy between *J* and the true number of estimated models summarizes the dependence between the estimates, and that authors report only the specification with the most significant results. In that case, the critical value that would undo the selection effect of the specification searching would be $F^{-1}\left(\sqrt[{J}]{1-\alpha }\right)$, where *F*(⋅) is the distribution function for the test statistic in question. This critical value is only bounded if it is possible to bound *J*. This gives rise to the emphasis by Sullivan, Timmermann, and White [16] on the ability of the analyst to specify the *universe* of tests conducted by a single researcher, or a field of researchers [6].

Third, a related point is the interpretation of any individual test statistic within a paper or within a literature. While a literature may collectively be biased by non-publication of null results, each individual test that is published may still show unbiased results on its own. In this case, our method in some sense requires a stricter test (lower type I error rate, and lower power) than intended. However, given the evidence that even individual papers with multiple experiments suffer from publication bias within the individual papers, we believe our method is still useful. (See the test for excess significance, developed in [50] and applied in [51] and [52].)

Fourth, as mentioned previously, one could modify our method and develop a different modified cutoff by choosing a different type I error rate among the selected sample of published results, not just the same *α* that is used in submission/publication to generate the satisificing model of publication bias. The reason we use *α* is for its intuitive appeal, keeping the level of false positives identical across the entire universe of tests and the selected sample of submitted tests, restoring the originally intended type I error rate.

Finally, we believe that our approach could be of use in fields where file-drawer effects are believed to be pervasive, by offering a simple rule of thumb to adjust after the fact for the rate of false positives. However, this method is unlikely to represent an active remedy to the problem of publication bias, as it may encourage a “t-ratio arms race,” whereby authors understand that editors are suspicious of t-ratios just above 2, and adjust their submission behavior accordingly. Authors would become ever more selective in their submissions as editors became ever more critical, *ad infinitum*. In light of these considerations, perhaps the best way to understand the result described above is as a useful rule of thumb to employ, assuming that few other people deviate from standard practice.

### Conclusion

In this paper, we have outlined a simple method for restoring the intended type I error rate of tests used by consumers of research (e.g., editors, readers) when producers of research (e.g., authors, editors) select results based on the statistical significance of tests, and where the selection follows a satisficing rule. The analysis shows that this selection effect in fact distorts the size of test statistics by approximately 50% and may be eliminated using adjusted critical values. These adjusted critical values are particularly simple to implement and require only a (detailed) table giving the distribution of the (unselected) test statistic under the null hypothesis. A leading example of the application of this result is two-tailed t-tests, where a test with type I error rate of 5 percent involves a critical value of 1.96. Distortions created by file drawer bias are adjusted for by using an adjusted critical value of 3.02. Samples of published social science research indicate that on average, across research fields approximately 30% of t-statistics are between the standard and adjusted critical values, and might thus be affected by the proposed method.
